# Development of a Novel Diagnostic Biomarker Set for Rheumatoid Arthritis Using a Proteomics Approach

**DOI:** 10.1155/2018/7490723

**Published:** 2018-11-26

**Authors:** Sora Mun, Jiyeong Lee, Mi-Kyoung Lim, You-Rim Lee, Chunhwa Ihm, Seung Hoon Lee, Hee-Gyoo Kang

**Affiliations:** ^1^Department of Senior Healthcare, BK21 Plus Program, Graduate School, Eulji University, Seongnam 13135, Republic of Korea; ^2^Department of Biomedical Laboratory Science, College of Health Sciences, Eulji University, Seongnam 13135, Republic of Korea; ^3^Division of Rheumatology, Department of Medicine, Eulji University School of Medicine, Daejeon 34824, Republic of Korea; ^4^Department of Laboratory Medicine, Eulji University Hospital, Daejeon 35233, Republic of Korea; ^5^Department of Neurosurgery, Eulji University School of Medicine, Daejeon 34824, Republic of Korea

## Abstract

**Background:**

Rheumatoid arthritis (RA) is an autoimmune disease that starts with inflammation of the synovial membrane. Studies have been conducted to develop methods for efficient diagnosis of RA and to identify the mechanisms underlying RA development. Blood samples can be useful for detecting disturbance of homeostasis in patients with RA. Nanoliquid chromatography-tandem mass spectrometry (LC-MS/MS) is an efficient proteomics approach to analyze blood sample and quantify serum proteins.

**Methods:**

Serum samples of 18 healthy controls and 18 patients with RA were analyzed by LC-MS/MS. Selected candidate biomarkers were validated by enzyme-linked immunosorbent assay (ELISA) using sera from 43 healthy controls and 44 patients with RA.

**Results:**

Thirty-eight proteins were significantly differentially expressed by more than 2-fold in healthy controls and patients with RA. Based on a literature survey, we selected six candidate RA biomarkers. ELISA was used to evaluate whether these proteins effectively allow distinguishing patients with RA from healthy controls and monitoring drug efficacy. SAA4, gelsolin, and vitamin D-binding protein were validated as potential biomarkers of RA for screening and drug efficacy monitoring of RA.

**Conclusions:**

We identified a panel of three biomarkers for RA which has potential for application in RA diagnosis and drug efficacy monitoring. Further, our findings will aid in understanding the pathogenesis of RA.

## 1. Introduction

RA causes inflammation of the synovial membrane that is surrounded by the joint capsule and is one of the intractable, rare diseases [1]. RA incidence is higher in women than in men, with women representing 70–80% of RA patients. It is assumed that a complex of causal factors, including genetic, environmental, and immunity-related factors, is involved in the development of RA. The World Health Organization has estimated that 1% of the world population and 0.7% of the total population of South Korea suffer from RA [2].

In 2010, the American College of Rheumatology/European League Against Rheumatism reported classification criteria for RA based on the extent of tenderness and swelling of the joints, serum levels of autoantibodies such as rheumatoid factor (RF) and anticitrullinated protein autoantibody (ACPA), levels of acute-phase reactants such as C reactive protein (CRP), erythrocyte sedimentation rate (ESR), and duration of symptoms. However, ACPA sensitivity is 60–75% and RF-positive rate is 60–70% [3, 4], which suggests that early diagnosis is very difficult and explains why RA has poor prognosis. It turns out that 70% of the joints is damaged within 2 years after onset of RA [5]. Additionally, for efficient drug dosing in patients with RA, assessment of disease activity is important. Disease activity score 28 (DAS 28) is used to measure disease activity of RA by several criteria such as CRP and ESR. However, because they are general markers of inflammation, they are not specific to RA.

Serum proteins can be powerful indicators of disease states. For example, proteins such as serum amyloid A, CRP, and calgranulin A, B, and C are differentially expressed in patients with RA as compared to healthy controls. Additionally, cytokines such as tumor necrosis factor (TNF)*α* and interleukin (IL)1*β*, IL6, IL13, and IL15 are increased in the serum of patients with RA [6, 7]. Therefore, biomarker discovery studies of serum samples for several diseases have been performed using proteomics approaches [8–10]. Liquid chromatography-tandem mass spectrometry (LC-MS/MS) is an efficient method to identify serum proteins that are differentially expressed in sera of patients as compared to healthy controls.

However, only few studies using clinical serum samples of patients with RA to confirm whether biomarker candidate proteins allow differentiating patients with RA from healthy controls have been conducted. And, there currently are limitations to the monitoring of disease activity and severity. Thus, using a proteomics approach, this study aimed to discover novel biomarkers for RA diagnosis and for measuring disease activity. We considered that new biomarkers allow monitoring disease activity to evaluate drug efficacy as well as diagnosis and would be helpful in the treatment of RA.

## 2. Materials and Methods

### 2.1. Serum Collection

This study was approved by the Institutional Review Board of the Korea Cancer Center Hospital (K-1408-002-069). Sera were collected from the Korea Institute of Radiological and Medical Sciences Radiation Biobank. For the proteomics experiment, samples from 18 healthy controls having RF < 18 IU/ml and from 18 patients with RA were used. For validation of candidate biomarkers by enzyme-linked immunosorbent assay (ELISA), 43 additional healthy controls and 44 additional patient samples were used. Basic demographic and clinical information on the study subjects is provided in Table 1.

### 2.2. Serum Depletion Using a Multiple Affinity Removal System LC Column

We followed the methods of Kang et al. 2018 [11], which is our previous work. The experiment was carried out in the same method from serum depletion to data analysis. High-abundance human serum proteins, such as albumin and immunoglobulin, were depleted by using a multiple affinity removal system LC column (human 6-HC, 4.6 × 50 mm; Agilent Technologies, Santa Clara, CA, USA). In brief, pooled sera were loaded onto the column and low-abundance proteins were eluted in buffer A (19808; Agilent Technologies, Wilmington, DE) and concentrated using a Pierce concentrator (7 mL/9K; Thermo Scientific, Rockford, IL, USA).

### 2.3. Sample Preparation and Tryptic Digestion

We followed the methods of Kang et al. 2018. [11] Briefly, the concentration of serum proteins was measured using the Bradford assay (Bio-Rad, Hercules, CA). Serum proteins (250 *μ*g) were reduced by treatment with 5 mM Tris(2-carboxyethyl)phosphine (Pierce, Rockford, IL, USA) at 37°C, 300 rpm, for 30 min. Next, the samples were treated for 1 h with 15 mM iodoacetamide (Sigma-Aldrich, St. Louis, MO, USA) at room temperature, 300 rpm, in the dark. Then, trypsin gold of mass-spectrometry grade (Promega, Madison, WI, USA) was used for digestion of the samples overnight at 37°C. Chemical reagents were cleared using a C18 cartridge (Waters, Milford, MA, USA).

### 2.4. Peptide Fractionation by OFFGEL Electrophoresis

The peptides were separated by their isoelectric point using OFFGEL fractionator with a 12-well setup (3100 OFFGEL Low Res Kit, pH 3–10; Agilent Technologies, Santa Clara, CA, USA). The protocol was followed as per the manufacturer's instructions. Micro Spin columns (Harvard Apparatus, Holliston, MA, USA) were used for chemical reagent clearance.

### 2.5. Nanoliquid Chromatography-Tandem Mass Spectrometry (LC-MS/MS)

Briefly, the peptide fraction was analyzed on a high-performance liquid chromatography-chip/quadrupole time-of-flight (Q-TOF) system (Agilent Technologies, Santa Clara, CA, USA), which included an Agilent 1200 series nano-LC system and an Agilent 6520 Q-TOF equipped with a chip cube interface. The sample was injected into a 360-nL enrichment column linked to a Polaris C18-A separation column (75 *μ*m × 150 mm, 3 *μ*m) at a flow rate of 0.3 *μ*L/min for 120 min. MS/MS analysis was conducted in the positive ionization mode of ion source. The drying gas temperature and flow were 300°C and 3 L/min, respectively. The mass-to-charge ratio (m/z) MS scan range was m/z 300–2400, and the MS/MS scan range was m/z 100–3000, at a rate of 4 spectra/s.

### 2.6. Protein Identification by Database Search

MS/MS spectra from triplicate runs were exported and searched against an MS/MS database to identify proteins and peptides on the Spectrum Mill MS Proteomics Workbench (Agilent Technologies). The search parameters were as follows: precursor mass tolerance, 20 ppm; product ion mass tolerance, 50 ppm; maximum ambiguous precursor charge, 3; 2 missed cleavages allowed; fully digested peptide by trypsin; fixed modification of carbamidomethyl cysteine; variable modifications of oxidized methionine; and N-terminal carbamylation. Autovalidation of MS/MS spectra was processed with default value. FDR threshold was 1.2.

### 2.7. Label-Free Quantification of Protein Expression

Relative quantification with 75^th^ percentile normalization was carried out using the Mass Profiler Professional software (Agilent Technologies). The 75th percentile normalization is used to adjust the 75th percentile intensity of each sample to equalize the total intensity. After normalization, fold change can be calculated by adjusting the abundance of the samples [12–14]. Relative amounts of proteins were quantified based on normalized intensities of proteins, and 2-fold differentially expressed proteins (DEPs) were filtered at* p *< 0.05.

### 2.8. Functional Analysis of DEPs

DEPs were assigned to process networks and pathway maps using GeneGo MetaCore v 6.15 (GeneGo, St. Joseph, MI, USA). Briefly, DEPs were uploaded into the GeneGo MetaCore software, and the 10 highest-ranking process networks and maps of DEPs were visualized as bar graphs.* p* values were calculated based on a hypergeometric distribution.

### 2.9. Validation of Identified Proteins by ELISA

ELISAs were conducted for 6 DEPs—gelsolin, haptoglobin, vitamin D-binding protein (VDBP), plasminogen, serum amyloid A-4 (SAA4), and retinol-binding protein 4—using commercial kits per the manufacturer's instructions (Cusabio, Wuhan, China).

### 2.10. Statistical Analysis

Scatter plots of ELISA results, receiver-operating characteristic (ROC) curves, and correlation analysis of 6 candidate biomarkers were carried out using GraphPad prism version 5.0 for Windows (GraphPad Software, La Jolla, CA, USA). Statistical analysis of ELISA results was conducted by independent* t*-tests using the Statistical Package for Social Sciences software for windows, version 16.0 (SPSS 16; SPSS, Chicago, IL, USA). Data are expressed as mean ± standard deviation (SD). Benjamini-Hochberg correction, an FDR-adjusted corrected* p* value, was conducted. A corrected* p* value < 0.05 was considered significant.

## 3. Results

### 3.1. Identification of Proteins by LC-MS/MS

**Figure 1:**
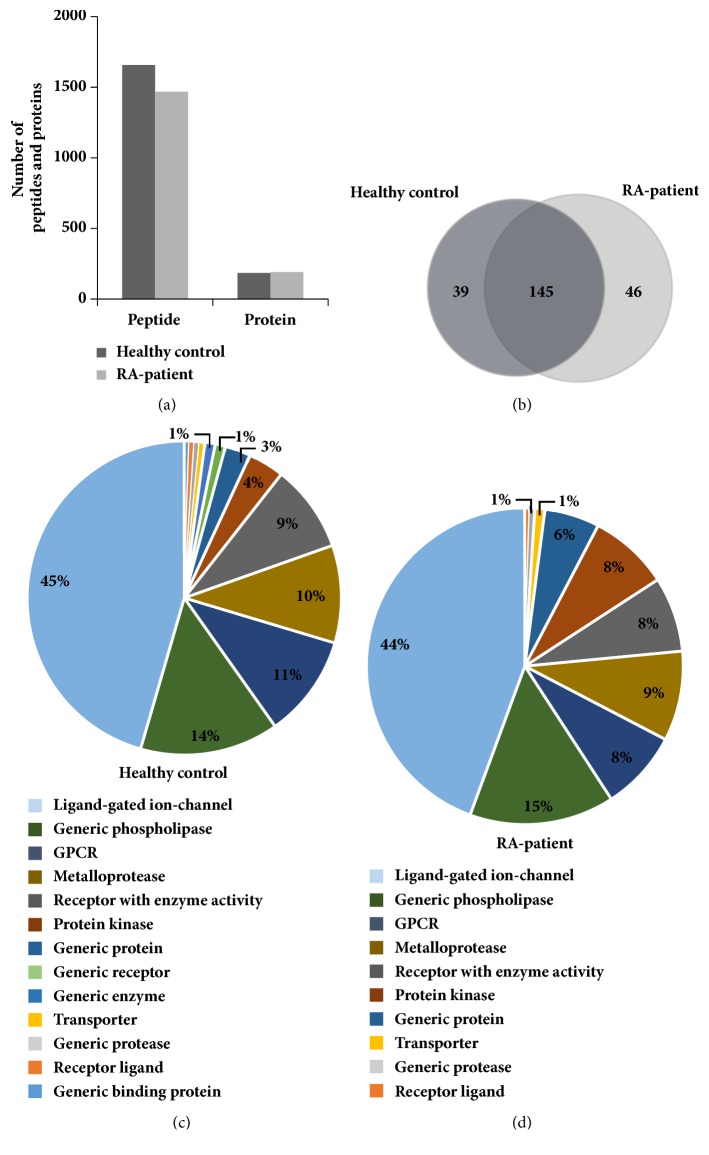
Identification of proteins by LC-MS/MS in sera of healthy controls and patients with RA. (a) Total numbers of peptides and proteins (at least 2 peptides) identified in the two groups. (b) Venn diagram of proteins identified in the two groups. Protein functions of identified proteins in (c) healthy controls and (d) patients with RA using GeneGo MetaCore software.

In total, 1657 peptides and 184 proteins were identified in healthy controls, and 1468 peptides and 191 proteins were identified in RA patients. Among these proteins, 39 were specific for healthy controls and 46 were specific for RA patients, while 145 were in common between both groups (Figures 1(a) and 1(b)). The results of functional analysis showed that ligand-gated ion-channels were 45% and 44% and generic phospholipases were 14% and 15% of the total proteins in healthy controls and patients with RA, respectively. Additionally, GPCR, metalloproteases, receptors with enzyme activity, protein kinases, and generic proteins, in order of increasing abundance, were identified in both healthy controls and patients with RA (Figures 1(c) and 1(d)). Meanwhile, generic receptors, generic enzymes, and generic binding proteins were identified only in the healthy controls (Figure 1(c)).

### 3.2. Analysis of Differentially Expressed Proteins

**Figure 2:**
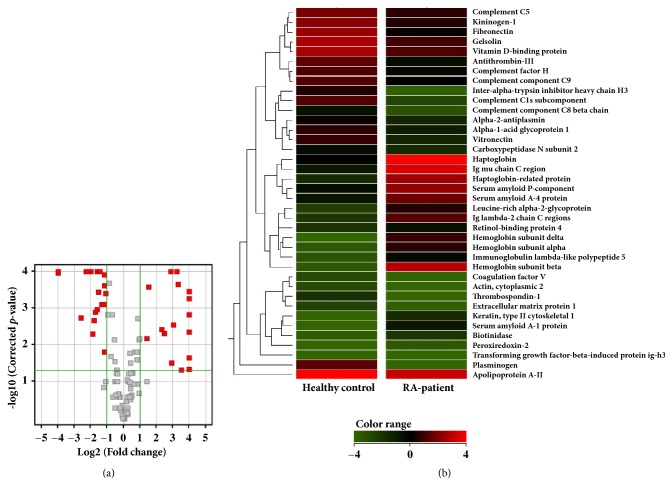
DEPs with statistical significance and cluster analysis of the proteins. (a) Volcano plot of >2-fold DEPs that are filtered by* p* value (*p *< 0.05). (b) Heat map visualizing the >2-fold DEPs.

**Figure 3:**
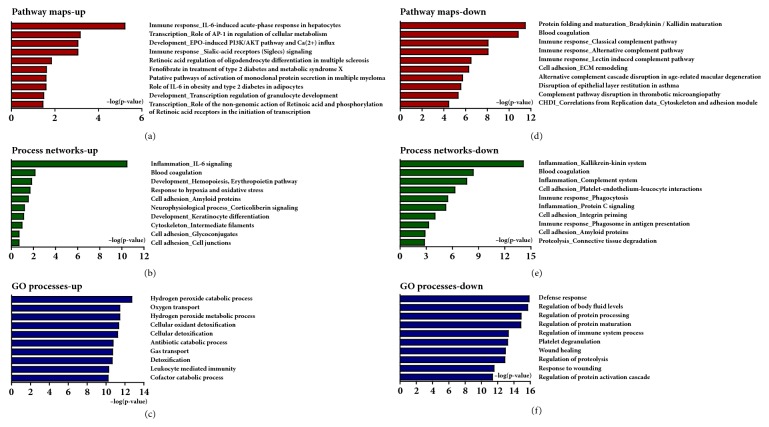
Pathway maps, process networks, and GO processes of >2-fold DEPs in patients with RA compared to healthy controls. (a) Significant pathway maps associated with >2-fold up-regulated proteins in patients with RA. (b) Significant process networks associated with >2-fold up-regulated proteins in patients with RA. (c) Significant GO processes associated with >2-fold up-regulated proteins in patients with RA. (d) Significant pathway maps associated with >2-fold down-regulated proteins in patients with RA. (e) Significant process networks associated with >2-fold down-regulated proteins in patients with RA. (f) Significant GO processes associated with >2-fold down-regulated proteins in patients with RA.

MPP software was used to quantify the expression ratios of identified proteins between healthy controls and patients with RA. Among the total proteins that were identified in healthy controls and patients with RA, 38 proteins were differentially expressed by more than 2-fold with statistical significance (*p *< 0.05) (Figure 2(a)). Of the 38 proteins, 16 were upregulated and 22 were downregulated in RA as compared to healthy controls. The heatmap shown in Figure 2(b) indicates the differential protein expression of these 38 proteins in healthy controls and RA patients. Functional analysis was conducted to identify the roles of the 38 DEPs in patients with RA. Pathway maps, process networks, and GO processes of DEPs are shown in Figure 3. The most significant pathway map of upregulated proteins was IL-6 induced acute-phase response in hepatocytes. The most significant pathway map of downregulated proteins was protein folding and bradykinin/kallidin maturation (Figures 3(a) and 3(b)). Plasminogen, plasmin, antithrombin, bradykinin, and KNG were involved in the second-most significant blood coagulation pathway, and Cls, C3a, C8beta, C5, C5b, and C9 were involved in the third-most significant classical complement pathway (Figure 3(b)), and the most significant process network of upregulated proteins was IL-6 signaling (Figure 3(c)). In addition, the most significant process network of downregulated proteins was the kallikrein-kinin system (Figure 3(d)). GO process analysis of upregulated and downregulated proteins showed that hydrogen peroxide catabolic process and defense response were activated in patients with RA (Figures 3(e) and 3(f)).

### 3.3. Selection and Validation of Biomarker Candidates for RA

**Figure 4:**
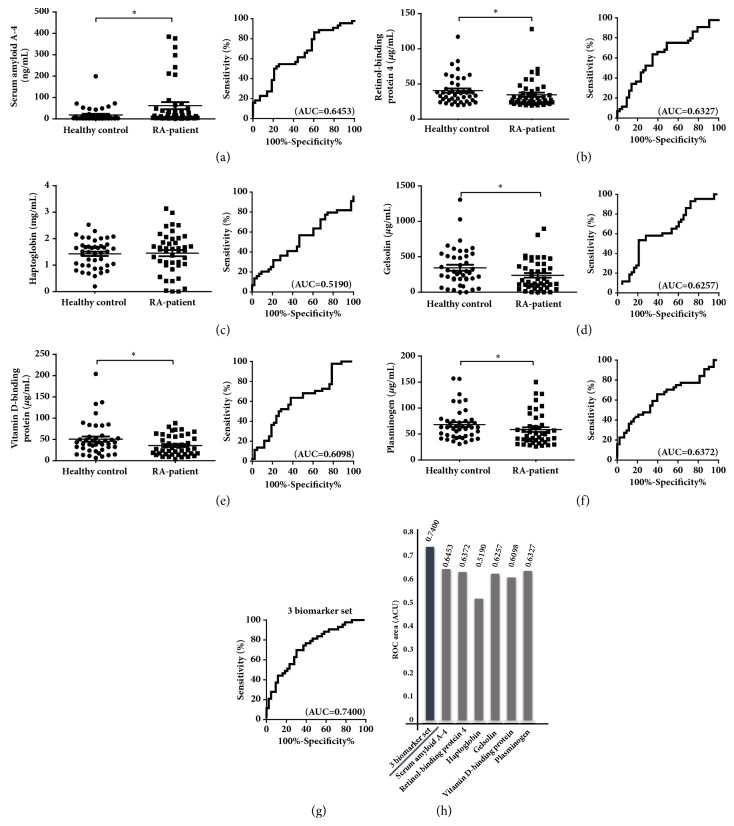
Validation of 6 selected biomarker candidates by ELISA and ROC curves for multibiomarkers. (a–f) The 6 selected biomarkers candidates were measured in sera from healthy controls and RA patients. (g) ROC curve analysis for 3-biomarker set, including SAA4, gelsolin, and VDBP to evaluate the ability of the biomarkers to distinguish patients with RA from healthy controls. (h) AUCs. Plots indicate individual protein abundances in healthy controls and RA patients. Data are shown as mean ± SEM. *∗p *< 0.05 (independent t-test). ROC curves for selected 6 biomarker candidates were generated to evaluate the ability of the biomarkers to distinguish patients with RA from healthy control.

**Figure 5:**
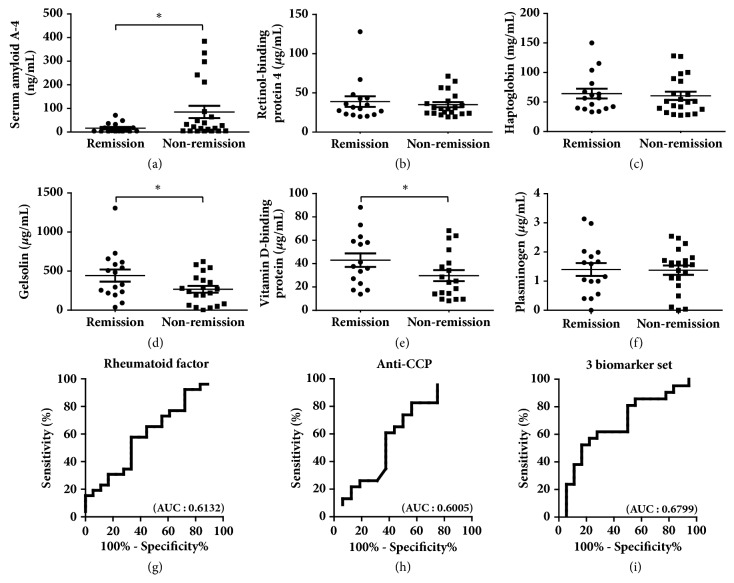
Differentiation of disease activity during drug treatment. (a–f) The 6 selected candidate biomarkers were measured in patients in remission and in patients in nonremission. (g-i) ROC curve analysis for rheumatoid factor, anti-CCP, and 3-biomarker set, including SAA4, gelsolin, and VDBP to evaluate the ability of the biomarkers to distinguish patients with RA from healthy controls. Plots indicate individual protein abundances in patients. Data are shown as mean ± SEM. *∗p* < 0.05 (independent t-test).

Of the 38 proteins, based on a literature search, we selected 6 biomarker candidates, including gelsolin, haptoglobin, VDBP, plasminogen, SAA4 protein, and retinol-binding protein 4, which are reportedly associated with RA [15–29]. Following studies for biomarker discovery, these markers may be used in the clinical field for diagnosing RA using MRM quantification. MRM is able to quantify target biomarker proteins. Therefore, proteins that could be analyzed by MRM were chosen as the final protein biomarker candidates in advance. Therefore, based on the data obtained from the SWATH relative quantification analysis, proteins that satisfy the following conditions were primarily selected. The list of proteins that filtered by fold change,* p* value, and literature review for biomarker selection is shown in Table S1. Gelsolin, VDBP, and plasminogen were downregulated, while haptoglobin, retinol-binding protein 4, and SAA4 were upregulated in patients with RA. ELISA is conventionally used to measure serum protein levels with high sensitivity and specificity. The selected 6 biomarker candidates identified by LC-MS/MS were confirmed by quantifying their concentrations in sera of patients and healthy controls using ELISA. Among the 6 proteins, 5 were statistically differentially expressed between healthy controls and patients with RA (Figure 4). SAA4 was upregulated in patients with RA, while plasminogen, retinol-binding protein 4, gelsolin, and VDBP were downregulated in patients with RA as compared to healthy controls. To evaluate the ability of the proteins to distinguish patients and healthy controls, ROC curve analysis was conducted. The areas under the ROC curves (AUCs) of SAA4, plasminogen, retinol-binding protein 4, gelsolin, and VDBP, plasminogen were 0.6453, 0.6327, 0.6257, 0.6098, and 0.6372, respectively (Figure 4). When ROC curves of SAA4, gelsolin, and VDBP were combined, the AUC was 0.7400 (Figure 4). These 3 multibiomarkers showed potential for monitoring RA severity and activity during drug treatment. Patients with RA were divided into two groups based on DAS 28 to evaluate the ability of biomarker candidates to monitor severity and activity of RA during drug treatment: patients who were in remission (DAS 28 < 2.6) and patients who were in nonremission (DAS 28 > 2.6). To verify the potential ability of these 3 multibiomarkers, existing biomarkers for RA, RF, and anti-CCP were evaluated by ROC analysis. As a result, the AUC of RF, anti-CCP, and 3 multibiomarkers were 0.6132, 0.6005, and 0.6799, respectively (Figure 5). Existing biomarkers had lower efficacy to monitor RA severity and activity than these 3 multibiomarkers. To confirm the sex-specific differences in the biomarker candidates, the candidate biomarkers were assessed in male and female patients. Patients were stratified according to sex. Serum amyloid A-4 and vitamin D-binding protein were higher in female subjects than in male subjects (Figure S1). To eliminate the effect of sex on marker performance, we compared the levels in female healthy controls and female patients with RA (Figure S2) or in male healthy controls and male patients with RA (Figure S3). The results showed that vitamin D-binding protein is specific for female subjects, and serum amyloid A-4 level increased in patients with RA compared with the healthy controls, regardless of the sex. Furthermore, retinol-binding protein 4, haptoglobin, gelsolin, and plasminogen did not show any sex-specific differences.

## 4. Discussion

We compared the levels of serum proteins between healthy controls and patients with RA using a nano-LC-MS/MS-based proteomics approach. As a result, 6 biomarker candidates were identified. The biomarker candidates were confirmed by ELISA, which showed that 5 biomarker candidates allowed differentiation of patients with RA from healthy controls. In addition, the combination of SAA4, gelsolin, and VDBP improved the ability to distinguish patients with RA from healthy controls, indicating that this 3-biomarker set could be useful to diagnose RA and is more powerful than using a single marker. Further, among the 5 proteins, SAA4, gelsolin, and VDBP could be monitored for assessing disease activity and severity during drug treatment.

GeneGo functional analysis of pathway maps and GO process networks revealed IL-6 signaling as the pathway map most strongly associated with the upregulated proteins in RA subjects compared to healthy controls. Moreover, bradykinin and kallidin maturation was the pathway map most strongly associated with the downregulated proteins in RA subjects compared to healthy controls. Bradykinin binds to bradykinin B2 receptor and causes inflammation and vasodilation [30]. Activation of bradykinin B1 receptor by kallidin causes pain perception and vasodilation [31]. Bradykinin is reportedly increased in RA patients and is related to RA development [32, 33]. In addition, pathway map analysis of downregulated proteins showed that the classical complement pathway involving C5a, C5, C5b, C8beta, and C9, which cause target cell lysis, was significantly activated in the patients. It is reported that the complement pathway is associated with the inflammatory response in RA [34]. GO process networks revealed that blood coagulation, involving plasminogen, plasmin, and coagulation factor V, was the second-most significant pathway activated in RA patients, which is consistent with previous findings that blood clotting-related proteins are associated with RA [35–37]. In accordance with our findings, in a previous study, levels of C1q-C4 were highly increased in the plasma of RA patients [38].

Gelsolin is an actin-binding protein that is associated with cell shape and motility [36, 39]. Gelsolin inhibits actin from depolymerizing and degrading the actin [40]. Lack of gelsolin in the serum is involved in acute and chronic inflammation, major trauma, and certain diseases [19, 41]. For example, in a previous study, plasma gelsolin was shown to be downregulated in acute liver failure, myocardial infarction, septic shock, and myonecrosis [42]. Plasma gelsolin is downregulated in patients with RA [15]. In RA, joints are destroyed through attack of the synovium by autoantibody [43]. Therefore, it is possible that gelsolin moves to regions of inflammation to attenuate the negative effects of the immune response. The movement of gelsolin to inflamed regions might cause a decrease in gelsolin in the blood [44]. Indeed, plasma gelsolin and gelsolin-actin complex have been identified in synovial fluid [19], which supports the hypothesis that gelsolin moves to the region of inflammation to regulate the negative effects of inflammation, such as microthrombosis, which involves actin filaments. In our study, gelsolin was downregulated in patients with RA as compared to healthy controls. Compared to previous studies, the extent of the reduction in gelsolin is remarkable in our study. Considering that the study subjects were over 50 years of age, it is suggested that gelsolin in the blood of elderly people is increasingly released to organs.

VDBP is a transport protein that transports biological molecules, including vitamin D, into effector cells [45]. VDBP is synthesized in the liver and has a molecular weight of 52–59 kDa [45]. Two major vitamin D metabolites are transferred to effector organs by VDBP: 25-hydroxyvitamin D (25(OH)-vitamin D; calcidiol) and 1,25-dihydroxyvitamin D (1,25(OH)2D; calcitriol) [46]. It is reported that vitamin D metabolites inhibit proinflammatory cytokines [45]. VDBP was shown to have a negative correlation with IL-6, suggesting the anti-inflammatory effect of VDBP on effector cells [47]. Further, VDBP plays a major role in scavenging actin, together with gelsolin, which is one of the defense mechanisms when homeostasis is destructed in diseases such as RA [48]. Actin exists in two major forms: the globular, monomeric form (G-actin) and the filamentous, polymeric form (F-actin) [45]. Damaged organs release F-actin into blood, which can block blood vessels and thus lead to microthrombosis [45]. To prevent these effects, actin-scavenging proteins such as gelsolin and VDBP need to remove the F-actin. It can be assumed that F-actin formation by damaged organs as well as the proinflammatory response is rampant in RA.

SAA4 is activated by cytokines such as IL-6 [49]. In our study, functional analysis of process networks showed that SAA4 is upregulated in patients with RA. In a previous study, SAA was reduced in patients with RA who were treated with tofacitinib, which targets IL-6 signaling [50]. Therefore, it is suggested that SSA might be an IL-6 signaling-related biomarker. SAA4 is reportedly more strongly expressed than CRP during inflammation [51]. Additionally, SAA is more efficient for confirming disease activity of RA as well as occurrence of inflammation than CRP [52]. Further, SAA is associated with AA amyloidosis [53], which is one of the complications in patients with RA [54]. Therefore, SAA might be a biomarker for the occurrence of AA amyloidosis in patients with RA.

Gelsolin, VDBP, and SAA4 might reflect the altered body environment, including local inflammation, which is followed by cellular damage. Moreover, these proteins can move freely between blood and organs. Therefore, gelsolin, VDBP, and SAA4 might be potent biomarker candidates for early diagnosis of RA. To assure the effectiveness of gelsolin, VDBP, and SAA4 as biomarkers to reflect the local inflammation environment, further investigation is needed. Future studies should also qualitatively analyze the three biomarker candidates in the synovial fluid and synovial membrane to confirm whether the three biomarker candidates are involved in local inflammation and reflect the local RA environment.

Based on the results, we suggest SAA4, gelsolin, and VDBP as candidate biomarkers for effective diagnosis of RA and drug efficacy monitoring. Nevertheless, there are some limitations in this study. First, we used 43 healthy subjects and 44 patients with RA for validation in ELISA. However, to validate the diagnosis efficacy of the 3-biomarker set, a larger cohort study has to be performed. Towards this, we have plans to conduct a further study with a large number of samples. Second, we evaluated whether the 3-biomarker set has the ability to distinguish disease activity between patients with remission and patients without remission. However, because it is important to precisely diagnose disease activity for adequate drug therapy, nonremission disease activity needs to be divided into subgroups including high, intermediate, and low disease activity. An assessment of patient's disease activity would help to treat patients with RA. Third, citrullinated peptides have been reported to be associated with RA. However, because citrullinated peptides have small mass and low abundance in samples, it is difficult to detect them by mass spectrometry. Nevertheless, considering their relevance to RA, they should be considered in further studies, even though they do not affect the overall mass analysis. Lastly, subjects who had no other diseases and no drug treatment history were selected as controls for the analysis of serum proteins for comparison between healthy controls and patients with RA. However, comparison between patients with rheumatic disease and rheumatoid arthritis is needed to confirm whether this 3-biomarker set has the ability to distinguish rheumatoid arthritis from other rheumatic diseases. We have plans to conduct the relevant experiments in future studies.

## 5. Conclusions

The 3-biomarker set comprising SAA4, gelsolin, and VDBP has the potential ability as a biomarker candidate for effective diagnosis of RA and drug efficacy monitoring. ELISA results confirmed that these three proteins are differentially expressed between healthy controls and patients with RA and allow differentiating disease activity during drug treatment. We expect that multibiomarkers comprising SAA4, gelsolin, and VDBP have stronger potential for diagnosis than single conventional biomarkers, such as RF and anti-CCP.

## Figures and Tables

**Table 1:** Basic demographic and clinical characteristics of the subjects.

	**Group**	**Number of subjects**	**RF (IU/mL)**	**Sex (Female/Male)**	**Anti-CCP (U/mL)**	**Age (Years)**
**LC-MS/MS**	**Healthy ** **c** **o** **n** **t** **r** **o** **l** **s** ^**a**^	18	6.5 ± 3.7	14/4	1.9 ± 8.1	65.1 ± 14.0
	**RA patients**	18	75.3 ± 94.6	14/4	124.5 ± 71.8	60.0 ±10.8

**ELISA (validation)**	**Healthy ** **c** **o** **n** **t** **r** **o** **l** **s** ^**a**^	43	5.8 ± 4.0	29/14	4.9 ± 24.8	60.1 ± 12.9
	**RA patients**	44	29.9 ± 9.7	37/7	96.1 ± 96.6	59.8 ± 9.0

RF, rheumatoid factor; LC-MS/MS, liquid chromatography-tandem mass spectrometry; ELISA, enzyme-linked immunosorbent assay.

^a^RF < 18 IU/mL.

Data are expressed as the mean ± SD.

## Data Availability

They are available from the corresponding author upon reasonable request.

## Supplementary Materials

Supplementary Materials Supplementary files include comparative analysis of level of biomarker candidates between females and males in patients with RA (Figure S1). In addition, levels of biomarker candidates were measured between female healthy controls and patients with RA (Figure S2) or male healthy controls and patients with RA (Figure S3). The list of selected proteins that were filtered by fold change,* p* value, and literature research is shown (Table S1).
