# Supplementary material for: Development of a Novel Diagnostic Biomarker Set for Rheumatoid Arthritis Using a Proteomics Approach
Source: Biomed Res Int. 2018 Nov 26;2018:7490723. doi: 10.1155/2018/7490723 (PMC6312602; doi:10.1155/2018/7490723)

Table S1. List of proteins filtered by fold change, p-value, and literature review.

Swiss-Prot ID	**Compound Name**	**FC > 2.0**	**FC>2.0, *p*<0.05[a]**	**Literature research**	**References[b]**
P04114	Apolipoprotein B-100				
P01024	Complement C3				
P0C0L5	Complement C4-B				
P0C0L4	Complement C4-A				
P01023	Alpha-2-macroglobulin				
P20742	Pregnancy zone protein				
P02647	Apolipoprotein A-I				
P01031	Complement C5	√	√		
Q14624	Inter-alpha-trypsin inhibitor heavy chain H4				
P00450	Ceruloplasmin				
P02751	Fibronectin	√	√		
P19823	Inter-alpha-trypsin inhibitor heavy chain H2				
P06727	Apolipoprotein A-IV				
P01011	Alpha-1-antichymotrypsin				
P01009	Alpha-1-antitrypsin				
P19827	Inter-alpha-trypsin inhibitor heavy chain H1				
P02790	Hemopexin				
P02649	Apolipoprotein E				
P05155	Plasma protease C1 inhibitor				
P06396	Gelsolin	√	√	√	[15], [18], [19]
P01008	Antithrombin-III	√	√		
P00738	Haptoglobin	√	√	√	[26]

P00739	Haptoglobin-related protein	√	√		
P00751	Complement factor B				
P08603	Complement factor H	√	√		
Q03591	Complement factor H-related protein 1		√		
P05546	Heparin cofactor 2		√		
P36955	Pigment epithelium-derived factor		√		
P10909	Clusterin		√		
P02748	Complement component C9	√	√		
P02774	Vitamin D-binding protein	√	√	√	[20], [21], [22]
P02787	Serotransferrin				
P04217	Alpha-1B-glycoprotein				
P01871	Ig mu chain C region	√	√		
P27169	Serum paraoxonase/arylesterase 1				
Q15166	Serum paraoxonase/lactonase 3				
P10643	Complement component C7				
Q06033	Inter-alpha-trypsin inhibitor heavy chain H3	√	√		
P08697	Alpha-2-antiplasmin	√	√		
P01042	Kininogen-1	√	√		
P00734	Prothrombin				
P03952	Plasma kallikrein				
P09871	Complement C1s subcomponent	√	√		
P12259	Coagulation factor V	√	√		
P00736	Complement C1r subcomponent				
Q9NZP8	Complement C1r subcomponent-like protein	√			
P06681	Complement C2				
P02766	Transthyretin				

P35858	Insulin-like growth factor-binding protein complex acid labile subunit				
P05543	Thyroxine-binding globulin				
P80108	Phosphatidylinositol-glycan-specific phospholipase D				
P29622	Kallistatin				
P05156	Complement factor I				
P02652	Apolipoprotein A-II	√	√		
P51884	Lumican				
P02750	Leucine-rich alpha-2-glycoprotein	√	√		
P13671	Complement component C6				
P01019	Angiotensinogen				
P02743	Serum amyloid P-component	√	√		
P07358	Complement component C8 beta chain	√	√		
P07996	Thrombospondin-1	√	√		
P25311	Zinc-alpha-2-glycoprotein				
P68871	Hemoglobin subunit beta	√	√		
P02042	Hemoglobin subunit delta	√	√		
P00747	Plasminogen	√	√	√	[23], [24], [25]
P01876	Ig alpha-1 chain C region				
P01877	Ig alpha-2 chain C region				
P15169	Carboxypeptidase N catalytic chain				
P43652	Afamin				
P02763	Alpha-1-acid glycoprotein 1	√	√		
P19652	Alpha-1-acid glycoprotein 2				
P04004	Vitronectin	√	√		
P22792	Carboxypeptidase N subunit 2	√	√		
P35542	Serum amyloid A-4 protein	√	√	√	[27], [28], [29]

O75882	Attractin		
P07357	Complement component C8 alpha chain	√	
O75636	Ficolin-3		
P02654	Apolipoprotein C-I	√	
P04264	Keratin, type II cytoskeletal 1	√	√
P35908	Keratin, type II cytoskeletal 2 epidermal		
P05452	Tetranectin		√
P04196	Histidine-rich glycoprotein		√
P04275	von Willebrand factor		√
Q96PD5	N-acetylmuramoyl-L-alanine amidase		√
O14791	Apolipoprotein L1		
P02765	Alpha-2-HS-glycoprotein		
Q9UK55	Protein Z-dependent protease inhibitor		
P02749	Beta-2-glycoprotein 1		
Q96KN2	Beta-Ala-His dipeptidase		
P01857	Ig gamma-1 chain C region		
P01860	Ig gamma-3 chain C region		
P01859	Ig gamma-2 chain C region		
P02671	Fibrinogen alpha chain		
P08185	Corticosteroid-binding globulin		
Q08380	Galectin-3-binding protein		
P22352	Glutathione peroxidase 3		
P02655	Apolipoprotein C-II		
Q9UHG3	Prenylcysteine oxidase 1		
P00915	Carbonic anhydrase 1		
P18428	Lipopolysaccharide-binding protein		
P04278	Sex hormone-binding globulin		
Q16610	Extracellular matrix protein 1	√	√

P13645	Keratin, type I cytoskeletal 10				
P07225	Vitamin K-dependent protein S				
P02656	Apolipoprotein C-III				
P06276	Cholinesterase				
P35527	Keratin, type I cytoskeletal 9				
P04180	Phosphatidylcholine-sterol acyltransferase				
P63261	Actin, cytoplasmic 2	√	√		
P43251	Biotinidase	√	√		
O00391	Sulfhydryl oxidase 1		√		
Q96IY4	Carboxypeptidase B2		√		
P69905	Hemoglobin subunit alpha	√	√		
P02775	Platelet basic protein				
P05090	Apolipoprotein D				
P08571	Monocyte differentiation antigen CD14				
Q9UGM5	Fetuin-B				
P55058	Phospholipid transfer protein				
P0CG05	Ig lambda-2 chain C regions	√	√		
B9A064	Immunoglobulin lambda-like polypeptide 5	√	√		
P02753	Retinol-binding protein 4	√	√	√	[16], [17]
P05154	Plasma serine protease inhibitor		√		
P02747	Complement C1q subcomponent subunit C		√		
Q92954	Proteoglycan 4		√		
P49908	Selenoprotein P				
P07360	Complement component C8 gamma chain				
O00533	Neural cell adhesion molecule L1-like protein				
O95445	Apolipoprotein M				
P02760	Protein AMBP				

P0DJI8	Serum amyloid A-1 protein	√	√
P00748	Coagulation factor XII		
P32119	Peroxiredoxin-2	√	√
P02768	Serum albumin		
P00740	Coagulation factor IX		
P08519	Apolipoprotein(a)		
P01880	Ig delta chain C region		
P01766	Ig heavy chain V-III region BRO		
P01781	Ig heavy chain V-III region GAL		
P01834	Ig kappa chain C region		
P11226	Mannose-binding protein C		
P07359	Platelet glycoprotein Ib alpha chain		
P02741	C-reactive protein		
P06702	Protein S100-A9		
A0A0B4J1X5	Immunoglobulin heavy variable 3-74		
Q9NQ79	Cartilage acidic protein 1		
P01619	Immunoglobulin kappa variable 3-20		
A0A0C4DH25	Immunoglobulin kappa variable 3-20		
P02746	Complement C1q subcomponent subunit B		
P04003	C4b-binding protein alpha chain		
P15144	Aminopeptidase N		
P13591	Neural cell adhesion molecule 1		
Q8WZ42	Titin		
P33151	Cadherin-5		
P05160	Coagulation factor XIII B chain		
P01614	Ig kappa chain V-II region Cum		
A0A087WW87	Immunoglobulin kappa variable 2-40		
P13796	Plastin-2		

O43157	Plexin-B1
A0A0C4DH38	Immunoglobulin heavy variable 5-51
P17936	Insulin-like growth factor-binding protein 3
P00742	Coagulation factor X
P06312	Immunoglobulin kappa variable 4-1
P09172	Dopamine beta-hydroxylase
P61769	Beta-2-microglobulin
Q14520	Hyaluronan-binding protein 2
P14151	L-selectin
A0A0A0MS15	Immunoglobulin heavy variable 3-49
Q9Y6R7	IgGFc-binding protein
P02788	Lactotransferrin
P41222	Prostaglandin-H2 D-isomerase
Q04756	Hepatocyte growth factor activator
P22891	Vitamin K-dependent protein Z
P04040	Catalase
P37802	Transgelin-2
P55056	Apolipoprotein C-IV
Q9P1Q0	Vacuolar protein sorting-associated protein 54
P40197	Platelet glycoprotein V
Q59EK9	RUN domain-containing protein 3A
P08294	Extracellular superoxide dismutase [Cu-Zn]
A0A0B4J1V0	Immunoglobulin heavy variable 3-15
P04206	Ig kappa chain V-III region GOL
Q7Z404	Transmembrane channel-like protein 4
Q86VP1	Tax1-binding protein 1
P19320	Vascular cell adhesion protein 1
Q15113	Procollagen C-endopeptidase enhancer 1

Q15582	Transforming growth factor-beta-induced protein ig-h3	√	√
Q13790	Apolipoprotein F		√
Q9Y6Z7	Collectin-10		√
P35443	Thrombospondin-4		√
P04070	Vitamin K-dependent protein C		√
P23083	Ig heavy chain V-I region V35		√
P08709	Coagulation factor VII		
A0A0B4J1U7	Immunoglobulin heavy variable 6-1		
P10720	Platelet factor 4 variant		
P03951	Coagulation factor XI		
Q92820	Gamma-glutamyl hydrolase		
Q6UX71	Plexin domain-containing protein 2		
P02679	Fibrinogen gamma chain		
P21333	Filamin-A		
Q9H8L6	Multimerin-2		
P01706	Immunoglobulin lambda variable 2-11		
P80748	Ig lambda chain V-III region LOI		
P01602	Immunoglobulin kappa variable 1-5		
P11597	Cholesteryl ester transfer protein		
O43866	CD5 antigen-like		
O75144	ICOS ligand		
P04433	Ig kappa chain V-III region VG (Fragment)		
P08637	Low affinity immunoglobulin gamma Fc region receptor III-A		
P13598	Intercellular adhesion molecule 2		
Q9UEU0	Vesicle transport through interaction with t-SNAREs homolog 1B		
P07737	Profilin-1		

Q9HDC9	Adipocyte plasma membrane-associated protein
O14786	Neuropilin-1
P07333	Macrophage colony-stimulating factor 1 receptor
Q9BRS2	Serine/threonine-protein kinase RIO1
P01717	Ig lambda chain V-IV region Hil
A0A0C4DH55	Immunoglobulin kappa variable 3D-7
P48058	Glutamate receptor 4
A0A075B6H9	Immunoglobulin lambda variable 4-69
P00918	Carbonic anhydrase 2
P05362	Intercellular adhesion molecule 1
Q96EE4	Coiled-coil domain-containing protein 126
P23470	Receptor-type tyrosine-protein phosphatase gamma
P55103	Inhibin beta C chain
Q01973	Tyrosine-protein kinase transmembrane receptor ROR1
Q14008	Cytoskeleton-associated protein 5

[a]) FDR was corrected by Benjamini-Hochberg correction or without any correction.

[b]) References supporting the association of 6 selected biomarkers with rheumatoid arthritis were cited in the manuscript.

Supplementary figure captions

Supplementary figure 1. Comparative analysis of the expression of biomarker candidates in female and male in patients with RA. (**a–f**) The selected 6 candidate biomarkers were measured in patients with RA. Patients were stratified according to sex. Plots indicate individual protein abundances in patients. Data are shown as mean ± SEM. *$p < 0.05$ (independent *t*-test). Healthy controls, n = 43, RA patients, n = 44.

Supplementary figure 2. Differences in the expression of biomarker candidates between female healthy controls and patients with RA. (**a–f**) The 6 selected candidate biomarkers were measured in female healthy subjects and patients with RA. Plots indicate individual protein abundances. Data are shown as mean ± SEM. *$p < 0.05$ (independent *t*-test).

Supplementary figure 3. Differences in the expression of biomarker candidates between male healthy controls and patients with RA. (**a–f**) The 6 selected candidate biomarkers were measured in male healthy subjects and RA patients. Plots indicate individual protein abundances. Data are shown as mean ± SEM. **$p < 0.001$ (independent *t*-test).

Supplementary figure 1

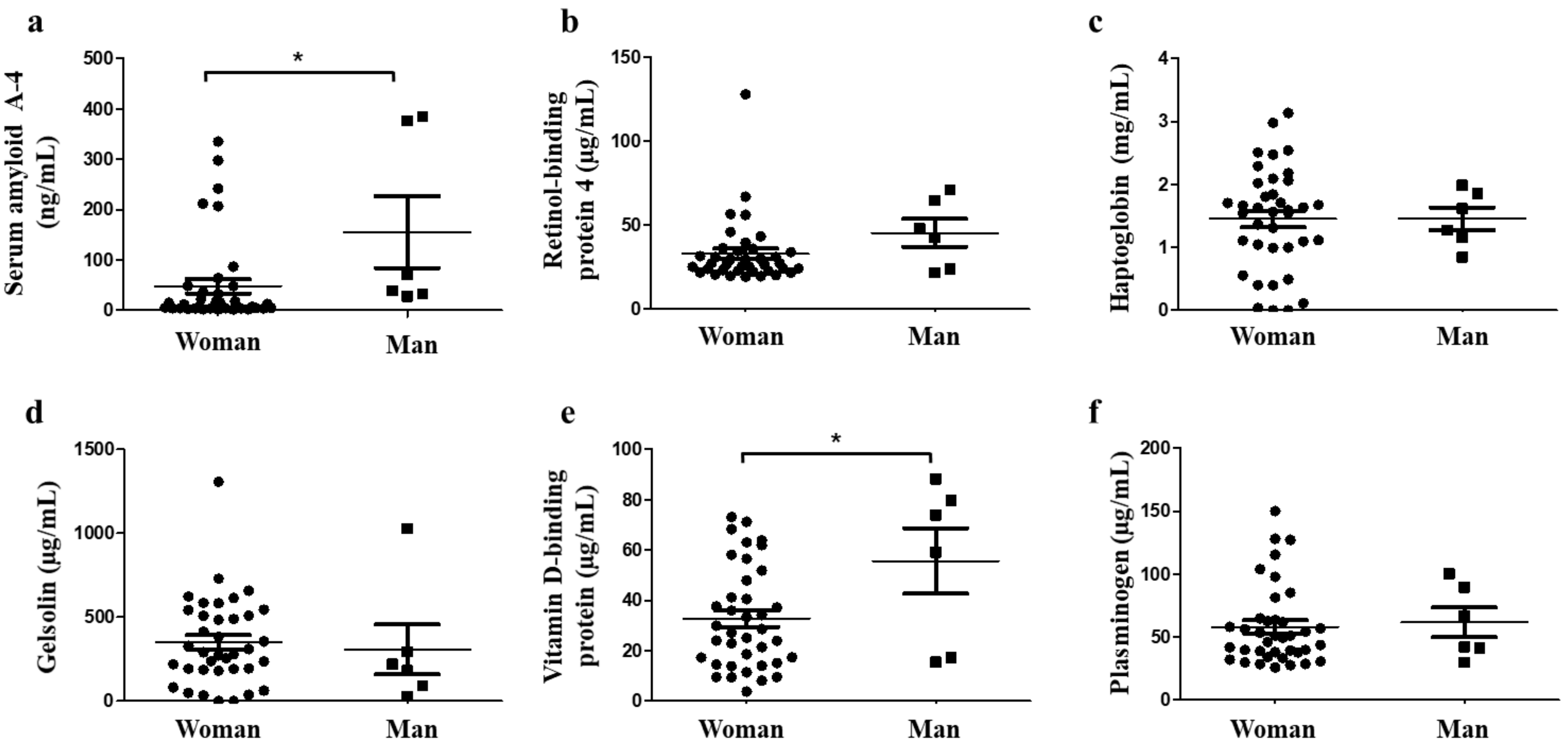

Supplementary figure 2

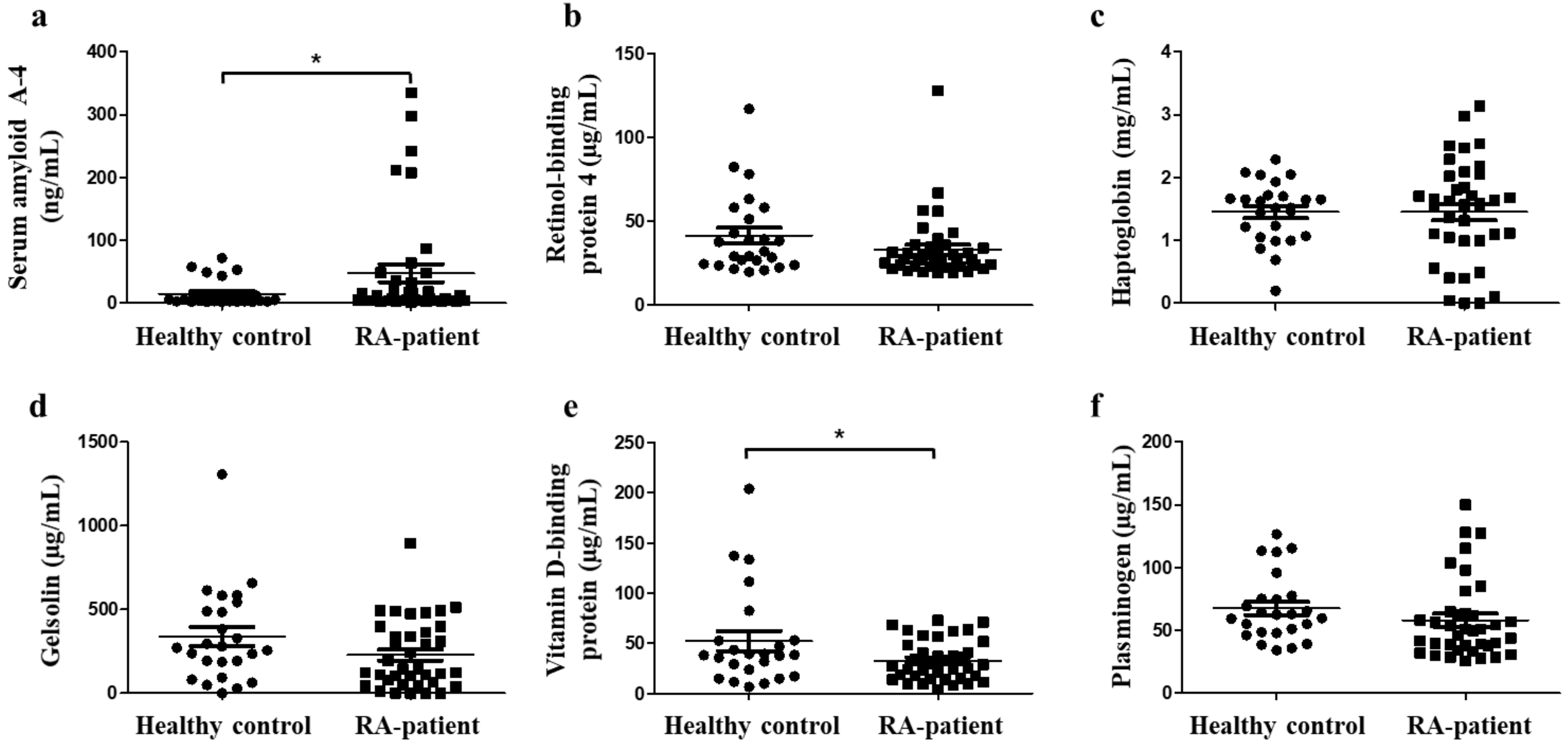

Supplementary figure 3

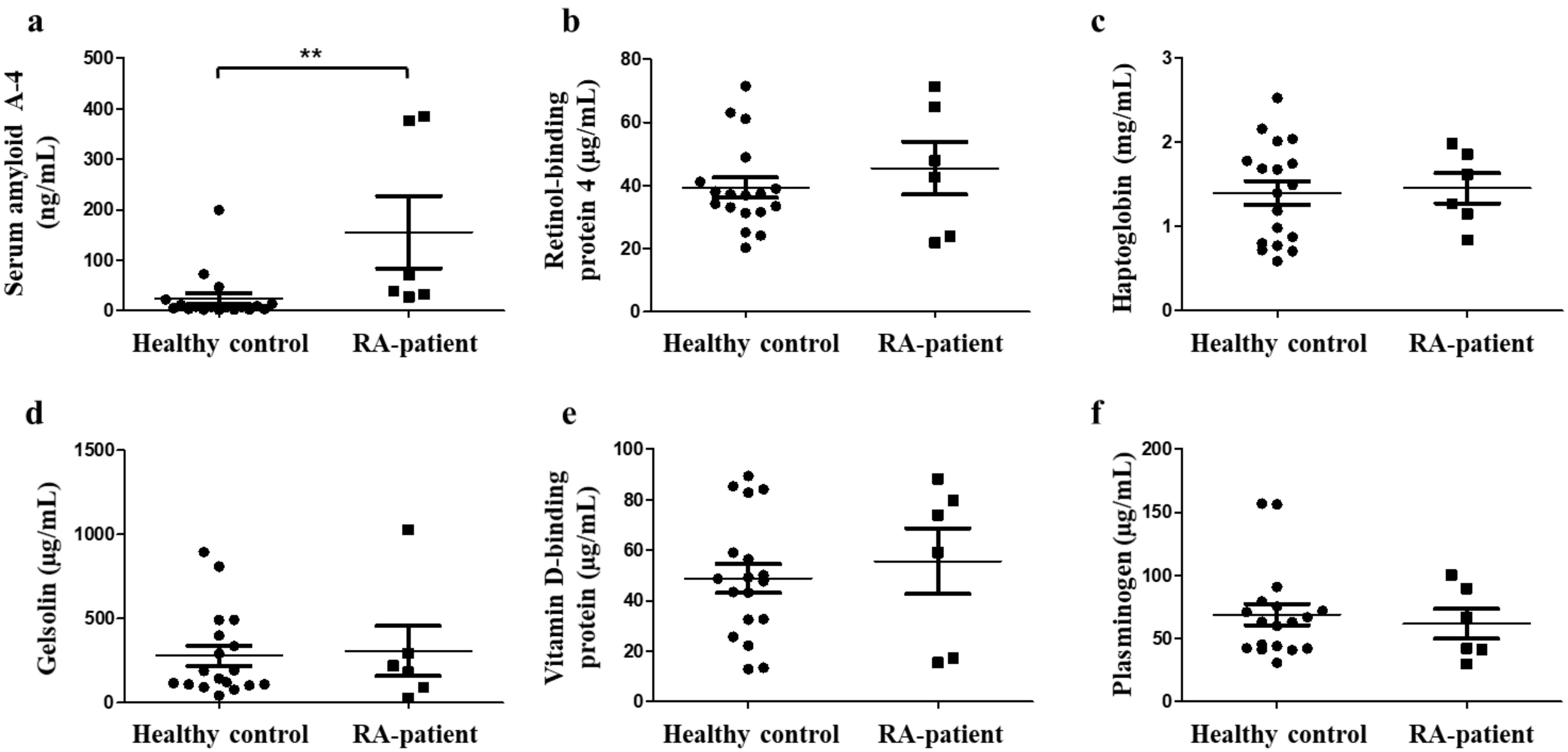

Supplement: Supplementary Materials — Supplementary files include comparative analysis of level of biomarker candidates between females and males in patients with RA (Figure S1). In addition, levels of biomarker candidates were measured between female healthy controls and patients with RA (Figure S2) or male healthy controls and patients with RA (Figure S3). The list of selected proteins that were filtered by fold change, p value, and literature research is shown (Table S1). [file 7490723.f1.pdf]
